# Comparison of Tooth Discoloration Induced by Calcium-Enriched Mixture and Mineral Trioxide Aggregate

**DOI:** 10.7508/iej.2016.03.005

**Published:** 2016-05-01

**Authors:** Armita Rouhani, Majid Akbari, Aida Farhadi-faz

**Affiliations:** a *Department of Endodontics, Dental Research Center, Dental School, Mashhad University of Medical Sciences, Mashhad, Iran; *; b * Department of Restorative Dentistry, Dental Research Center, Mashhad University of Medical Sciences, Mashhad, Iran; *; c * Department of Endodontics, Kerman University of Medical Sciences, Kerman, Iran*

**Keywords:** Calcium-Enriched Mixture, Dental Cements, Mineral Trioxide Aggregate, Tooth Discoloration, Vital Pulp Therapy

## Abstract

**Introduction:**

The aim of this *in vitro *study was to evaluate the tooth discoloration induced by calcium-enriched mixture (CEM) cement and mineral trioxide aggregate (MTA).

**Methods and Materials:**

Forty five endodontically treated human maxillary central incisors were selected and divided into three groups (*n*=15) after removing the coronal 3 mm of the obturating materials. In the MTA group, white MTA plug was placed in pulp chamber and coronal zone of the root canal. In CEM cement group, CEM plug was placed in the tooth in the same manner. In both groups, a wet cotton pellet was placed in the access cavity and the teeth were temporarily sealed. After 24 h the teeth were restored with resin composite. In the negative control group the teeth were also restored with resin composite. The color change in the cervical third of teeth was measured with a colorimeter and was repeated 3 times for each specimen. The teeth were kept in artificial saliva for 6 months. After this period, the color change was measured again. Data were collected by Commission International de I'Eclairage's L*a*b color values, and corresponding *ΔE* values were calculated. The results were analyzed using the one-way ANOVA and post-hoc Tukey’s test with the significance level defined as 0.05.

**Results:**

There was no significant differences between CEM group and control group in mean discoloration. The mean tooth discoloration in MTA group was significantly greater than CEM and control groups (*P*<0.05).

**Conclusion:**

According to the result of the present study CEM cement did not induce tooth discoloration after six months. Therefore it can be used in vital pulp therapy of esthetically sensitive teeth.

## Introduction

Biomaterials are used in many endodontic treatments, including repair of tooth perforation, as root end filling material, treatment of teeth with open apices and as pulp capping agents. One of these biomaterials which is being widely used is mineral trioxide aggregate (MTA). MTA is a biocompatible material with profound sealing properties that make it suitable for sealing root perforations [1-5]; and root end fillings [1, 6]. MTA is also used as a pulp capping agent and treatment of teeth with open apices [7]. However, one of the major drawbacks of MTA is tooth discoloration potential; which can be produced by both white and grey formulations [7-9]. 

Many studies have confirmed tooth discoloration potential of MTA. Kohli *et al.* [10] evaluated coronal tooth discoloration by various bioceramic cements and stated that both white and gray formulations of MTA caused significant discoloration in time. Kang *et al.* [11] also evaluated color change of teeth after using different MTA based materials and showed ProRoot MTA and Angelus MTA caused distinct color changes. 

Calcium-enriched mixture (CEM), is another biomaterial [12], with high biocompatibility and low cytotoxicity, antibacterial

properties and good sealing ability [13-15]. Its clinical applications are similar to those of MTA, including root-end filling, pulp capping, pulpotomy of primary and permanent teeth, repair of tooth perforations and vital pulp therapies [15-19].

One of the major concerns in using dental materials especially in aesthetically strategic areas is potential of tooth discoloration. The aim of this *in vitro *study was to evaluate the tooth discoloration potential of CEM cement in comparison with MTA.

## Materials and Methods

Forty five freshly extracted single-rooted human maxillary central incisors were used in this study. All specimens were clinically and radiographically examined for the absence of caries, cracks, restoration, discoloration and calcification. External surfaces of the teeth were cleaned with curette and prophylactic brushes and stored in a physiologic saline solution until usage.

After preparation of the access cavity and removing the pulp tissue by barbed broaches (Dentsply, Tulsa Dental, Tulsa, OK, USA), working length was determined visually after reducing 1 mm from length of a #10 K-file (Dentsply, Tulsa Dental, Tulsa, OK, USA) inserted into the canal after emergence of its tip from the apical foramen. 

Cleaning and shaping of each canal was done by using K-files (Dentsply, Tulsa Dental, Tulsa, OK, USA) and Gates-Glidden drills (Dentsply, Tulsa Dental, Tulsa, OK, USA) in a step-back manner and the apical area was prepared up to #40. A 2.5% concentration of sodium hypochlorite was used for irrigation of root canals. The root canals were dried with paper points and a #40 master gutta-percha (Aryadent, Tehran, Iran) was placed in the canal and confirmed radiographically. Obturation of each tooth was carried out using lateral compaction of gutta-percha and AH-Plus sealer (Dentsply, De Trey, Konstanz, Germany). The cutting of gutta-percha was done 3 mm below the orifice and the obturating material was compacted vertically. The remnants of sealer and gutta-percha were completely removed from the pulp chamber.

Then the teeth were randomly divided into three groups (*n*=15) including one negative control group. In MTA group, a white MTA plug (Angelus, Londrina, PR, Brazil) was placed in pulp chamber and the root canal space below the orifice. In CEM cement group, CEM cement plug (Yektazist Dandan, Tehran, Iran) was placed in the tooth in the same manner as MTA group. In both MTA and CEM cement groups, the materials were mixed according to the manufacturers’ instructions and a wet cotton pellet was placed in the access cavity and coronal seal was achieved using Coltosol (Coltene, Altstatten, Switzerland) for 24 h. After that, temporary filling and cotton pellet was removed and the materials were checked for setting. Then the teeth were restored with resin composite (Z100, 3M ESPE, MN, USA). In the negative control group the access cavity of the teeth were restored with resin composite. 

A colorimeter (Minolta CR-300; Minolta Co, Osaka, Japan) was used relative to standard illuminant with a white background to measure the color of each specimen in a standardized condition according to the CIE LAB (Commission International de l’Eclairage L*a*b) color space system. In this system, the *L* axis indicates the value that showed the degree of lightness [ranges from 0 (black) to 100 (white)], whereas *a* plane represents the degree of red or green [+*a* (red) and -*a* (green)] and *b* plane corresponds with the degree of yellow or blue [+*b* (yellow) and -*b* (blue)] within the sample. To position the tip of the colorimeter in the same location on each specimen, a silicon rubber mold was prepared. The colorimeter was calibrated on white calibration plate according to the manufacturer’s instruction. The color of the cervical third of the teeth was assessed three times and the mean value was considered as the final measurement at the baseline examination. The teeth were then kept in an incubator 37^°^C in artificial saliva for 6 months, whereas artificial saliva was replenished each week. After this period, color assessment was made using the colorimeter in the manner described for baseline readings. The calculation of the discoloration (ΔE*) between the two color measurements is as follows: ΔE*=[(ΔL*)2+(Δa*)2+(Δb*)2]1/2. The human eye cannot perceive color difference between two specimens (*ΔE*) values less than 1. *ΔE* values between 1 and 3.3 represent a clinically acceptable range [20]. *ΔE* values of 3.3 and higher are reported to be unacceptable for human eyes in clinical conditions [21] thus 3.3 has been used as the upper limit in some studies based on the perceptibility of color differences.[22-25] 

Preliminary analysis with Kolmogorov-Smirnov test was used to confirm the normal distribution of data. The results were analyzed using the one-way ANOVA and post-hoc Tukey’s test with the significance level set at 0.05.

## Results

The changes of color determinants from the baseline in the study groups are shown in Table 1. The mean±SD values of color change and descriptive statistics of the discoloration after 6 months in all groups are presented in Table 2. 

There was no significant differences between CEM cement group and control group in mean discoloration value. The mean tooth discoloration in MTA group was significantly more than CEM cement and control groups (*P*=0.000)

**Table 1:** Changes of color determinants in the study groups

**Groups**	***ΔL***	***Δa***	***Δb***	***ΔE***
**MTA**	2.98	1.14	1.58	3.56
**CEM cement**	2.02	0.89	1.06	2.45
**Control**	1.86	0.75	0.91	2.20

**Table 2:** The mean±SD of *ΔE* in study groups

**Groups (N)**	**Mean±SD**
**MTA (15)**	3.5613±0.70590
**CEM cement(15)**	2.4507±0.51825
**Control (15)**	2.2007±0.38524
**Total (45)**	2.7376±0.46675

## Discussion

Many studies have pointed to tooth discoloration as one of the major drawbacks of MTA [7-9]; which has been attributed to the presence of iron and manganese in the formulation of MTA [7]. The concentration of carborundum (Al_2_O_3_), periclase (MgO) and FeO has been lowered in white MTA compared to grey MTA but these metal oxides are still present in white preparations [26]. Marciano *et al.* [27] examined white Angelus MTA and observed that the reaction of dentin matrix collagen with bismuth oxide content in MTA caused a grayish discoloration of the tooth. Berger *et al.* [28] also reported combination of bismuth oxide with other chemical moieties as the primary cause of tooth discoloration.

Similarly EndoCem Zr (Maruchi, Wonju, Korea) and Retro MTA (BioMTA, Seoul, Korea) (both containing zirconium oxide) caused less discoloration than ProRoot MTA and Angelus MTA (which contain bismuth oxide) [11]. 

CEM cement, on the other hand contains CaO, SO_3_, P_2_O_5_, and SiO_2_ [29]. Despite the presence of metal oxides in CEM cement, in the present study, there was no tooth discoloration by this material after six months. However, all samples in MTA group showed significantly more discoloration than CEM group and control group after this period. Jacobovitz and de Lima [9] also observed grey discoloration with internal inflammatory root resorption in teeth treated with white MTA after 20 months. Akbari *et al.* [8] showed that both white and grey MTA placed below the root canal orifice, induced crown discoloration especially in the cervical area of teeth after six months. Jang *et al.* [30] also observed both ProRoot and Angelus MTA caused discoloration at the MTA-dentin interface and on the interior surface of dentin. 

Felman and Parashos [31] restored teeth with white MTA and found tooth discoloration in the crowns of samples which was prominent in the cervical third of teeth. It is clear that, in cases of vital pulp therapies of anterior teeth, such as apexogenesis, MTA is the material of choice due to its excellent biocompatibilities; but in most cases, discoloration of the tooth makes it a challenging treatment. 

CEM cement is a hydrophilic endodontic biomaterial. It has pH values similar to MTA [10]. Samiee *et al.* [32] showed both MTA and CEM cement had similar favorable biological response in furcation perforation repair and induced formation of cementum-like hard tissue and due to setting in wet environment, they are ideal as a perforation repair material. Two randomized controlled trials showed favorite outcomes following using CEM cement after vital pulp therapies of primary molar teeth [16, 32]. CEM cement also has good sealing ability as a root-end filling material [15]. Razmi *et al.* [33] showed both MTA and CEM cement has antibacterial effects against *E.* *faecalis*. Kangarlou *et al.* [34] also evaluated antifungal effects of CEM cement and found that it has fungicidal effects against *Candida albicans* even in low concentration. Similar to the result of this study, Eghbal *et al.* [35] evaluated color stability of white MTA and CEM cement and found that after irradiation, color stability of MTA was inferior to CEM cement samples.

Color measurement has several techniques and in the present study the quantitative technique was used. The quantitative measurement of color difference (ΔE*) with a colorimeter confers advantages such as repeatability, sensitivity and objectivity, despite some limitations [36, 37].

## Conclusion

According to the results of this study, CEM did not have discoloration properties unlike other endodontic materials like MTA. CEM cement can be advocated as an endodontic biomaterial in esthetically sensitive teeth.
